# Experimental evolution gone wild

**DOI:** 10.1098/rsif.2015.0056

**Published:** 2015-05-06

**Authors:** M. Scheinin, U. Riebesell, T. A. Rynearson, K. T. Lohbeck, S. Collins

**Affiliations:** 1 GEOMAR Helmholtz Centre for Ocean Research Kiel, Düsternbrooker Weg 20, Kiel, Germany; 2 Tvärminne Zoological Station, University of Helsinki, J. A. Palménin tie 260, Hanko, Finland; 3 Graduate School of Oceanography, University of Rhode Island, Narragansett, RI, USA; 4 Institute of Evolutionary Biology, University of Edinburgh, Ashworth Laboratories, The King's Building's, West Mains Road, Edinburgh, UK

**Keywords:** *Skeletonema*, diatom evolution, *in situ* mesocosms, carbon dioxide, ocean acidification, experimental evolution

## Abstract

Because of their large population sizes and rapid cell division rates, marine microbes have, or can generate, ample variation to fuel evolution over a few weeks or months, and subsequently have the potential to evolve in response to global change. Here we measure evolution in the marine diatom *Skeletonema marinoi* evolved in a natural plankton community in CO_2_-enriched mesocosms deployed *in situ*. Mesocosm enclosures are typically used to study how the species composition and biogeochemistry of marine communities respond to environmental shifts, but have not been used for experimental evolution to date. Using this approach, we detect a large evolutionary response to CO_2_ enrichment in a focal marine diatom, where population growth rate increased by 1.3-fold in high CO_2_-evolved lineages. This study opens an exciting new possibility of carrying out *in situ* evolution experiments to understand how marine microbial communities evolve in response to environmental change.

## 1. Introduction

Experimental evolution is a method that uses replicate populations, in controlled environments, to measure evolution in real time [1]. The power of this approach is that first, it produces generalizable results that further our understanding of how natural selection and evolution work—it allows us to uncover the rules that evolution plays by. This is partly because analyses focus on fitness, which is what natural selection acts on, regardless of particular genetic, epigenetic and phenotypic changes that underlie fitness shifts. Second, the experimenter manipulates the environment and uses replicate populations, so environmental changes can be linked causally to evolutionary responses [2]. However, experimental evolution has largely been confined to laboratory populations, and there are few experimental evolution studies on natural microbial populations *in situ*. Here, we show that marine mesocosms can be used for microbial evolution experiments by measuring evolution in a marine diatom in CO_2_-enriched marine mesocosms. This provides a link between laboratory evolution experiments and natural populations by using enclosures that are tractable, controllable and offer replication, but which also keep focal species in the context of a more natural community and habitat than is possible in the laboratory.

One limitation of experimental evolution stems from the same characters that give it its power: such replication, control and tractability can usually only be achieved under laboratory conditions, leading to a trade-off between uncovering general evolutionary mechanisms and understanding how they apply in complex natural environments, which in turn limits our understanding of how natural populations evolve in response to particular environmental drivers [2,3]. To make predictions about evolution in natural populations, it is vital that we link laboratory experiments to field studies. We propose that the most obvious way to do this is by conducting evolution experiments *in situ*. This requires the following criteria to be met: a starting population needs to be divided among independent replicate control and treatment environments. In addition, to measure evolution directly, rather than infer it from population genetics, the focal organisms need to reproduce quickly enough (or experience enough selective mortality) to measure heritable changes in fitness or genotype frequencies over an experiment. Previous evolution experiments in natural populations have taken advantage of natural replicate selection environments, such as stream systems where fish populations can be transplanted, for example to study predator/prey evolution [4]. However, this relies on finding fortuitous replicate environments (streams). We show that mesocosm enclosures fulfil the above criteria and can be coopted for microbial experimental evolution.

Marine mesocosms are commonly used to study community-level responses to environmental changes such as CO_2_ enrichment [5,6]. They are analogous to laboratory evolution experiments in that replicate mesocosms enclose random samples of the same aquatic community, which are subjected to an environmental change (e.g. CO_2_ enrichment) or not (control mesocosms). Because the mesocosms are closed, biotic or abiotic changes within them can be causally linked to the environmental manipulation. Here, we measure the evolutionary response to CO_2_ enrichment in a focal species of marine diatom during a mesocosm experiment.

## 2. Material and methods

### 2.1. Mesocosm set-up

**Figure 1.**
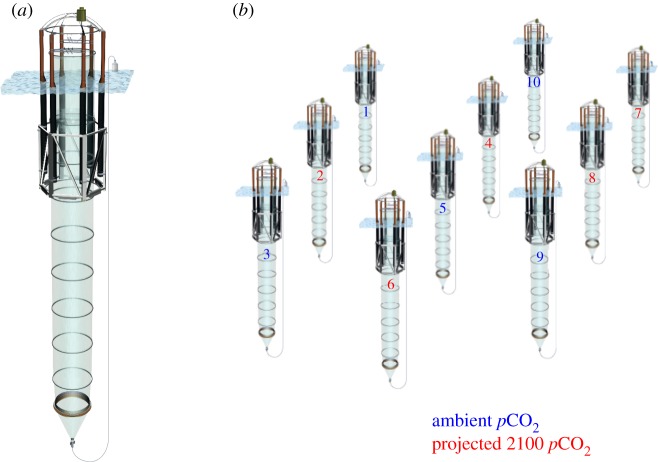
(*a*) Single mesocosm unit, consisting of flotation frame, mesocosm bag and sediment trap. (*b*) Experimental set-up, consisting of 10 mesocosm units, of which five are kept at ambient *p*CO_2_ level of approximately 400 µatm (control, numbers 1, 3, 5, 9 and 10) and five are manipulated to yield a *p*CO_2_ level of approximately 1000 µatm projected for the end of this century in the case of unabated CO_2_ emissions (numbers 2, 4, 6, 7 and 8). (Online version in colour.)

A mesocosm study was conducted in the Gullmar Fjord on the west coast of Sweden (58°15.9′ N, 11°28.9′ E) in the framework of the German national project on ‘Biological Impacts of Ocean Acidification’ (BIOACID) (figure 1). Detailed information about the mesocosm design and experimental application is provided in [7]. Briefly, 10 mesocosms were deployed in the Gullmar Fjord by R/V Alkor on 29 January 2013. The 18 m long enclosure bags were filled with fjord water after the retreat of sea-ice on 7 March, well before the start of the spring phytoplankton bloom. Each mesocosm bag enclosed about 55 m^3^ of fjord water, including the natural plankton community present at the time of closure. While five mesocosms were kept untreated as controls, the carbonate chemistry in the remaining five mesocosms was manipulated to establish elevated *p*CO_2_ at an initial level of 1100 µatm (for details on the manipulation approach, see Riebesell *et al.* [7]). The mesocosms were sampled every second day from 8 March to 28 June, covering a period of 107 days. In total, 45 parameters were measured in all mesocosms, providing a detailed overview of the environmental conditions in the mesocosms and the development of the enclosed plankton community.

### 2.2. Cell isolation

We attempted to isolate cells from all 10 mesocosms. Viable samples were obtained from five of the high CO_2_ mesocosms and three of the control mesocosms. Our experiment required isolating the same species of diatom from the majority of the mesocosms. *Skeletonema marinoi* was present in most mesocosms by the end of the experiment, although densities were low. Individual chains of *S. marinoi* were isolated from mesocosms and used to obtain monoclonal cultures. Samples were taken after the end of the mesocosm experiment (days 107–111) because net hauls would have been disruptive to other studies taking place at the same time. Plankton nets (mesh size 10 µm) were hauled over the whole depth of each mesocosm at 0.5 m s^−1^. Four hauls were done per mesocosm; total volume covered per mesocosm was 1064 l. Nets were emptied into 20 l carboys prefilled with filtered (0.2 µm) mesocosm water. Sampling gear was sterilized between mesocosms by soaking in 80% ethanol and rinsing in Milli-Q water. The carboys were stored in the dark at 10°C for 1–4 days before isolations were conducted. Cells were isolated on a 5 µm mesh, and collected by rinsing the mesh with sterile-filtered mesocosm water into Petri dishes.

*Skeletonema marinoi* was visually identified using an inverted light microscope (Leica DMIL). No other species of *Skeletonema* have been reported in the Gullmar Fjord (Swedish Meterorological and Hydrological Institute database SHARK/Svenskt HavsARKiv). Individual chains of cells were isolated with a 10 µl pipette and placed in a single well of a 24 well plate in 1 ml of f/8 medium [8] made from sterile-filtered water pooled from all 10 mesocosms. Growing isolates were transferred to 50 ml culture flasks containing 20 ml of media. Cultures were grown at 10°C at 100 µmol photons m^–2^ s^–1^ on a 12 L : 12 D cycle, and 20 µl of culture was transferred into fresh media every 5 days. This was done in the Sven Lovén Centre for Marine Sciences in Kristineberg, Sweden. There was a laboratory contamination event approximately five weeks after cells were isolated, and cultures were cleaned by reisolating *S. marinoi* cells. Surviving uncontaminated (cleaned) isolates are used for all work below. For the results reported in this manuscript, the final numbers of isolates from the three control mesocosms were 5, 3 and 7 isolates. The final numbers of isolates from the high CO_2_ mesocosms were 3, 8, 0, 9 and 11 isolates.

### 2.3. Laboratory culture conditions

Cultures were moved to the GEOMAR Helmholtz Centre for Ocean Research Kiel, Germany, for growth assays, and acclimated over 20 days to f/8 medium with artificial seawater [9] by replacing half the medium at each transfer over four transfers. In the final medium, 2% of the volume was seawater from the mesocosms, salinity was 35, and total alkalinity (TA) was adjusted to 2380 µEq l^−1^ using sodium bicarbonate. Cultures were kept in incubators (RUMED Light Thermostat Type 1201) at 5°C with 90–100 µmol photons m^–2^ s^1^ on a 12 L : 12 D cycle. Temperature was changed in one step. This temperature is closer to the temperature of the Gullmar Fjord during the mesocosm experiment; it was not possible to culture at 5°C at the Sven Lovén Centre. Growth rates were measured under these conditions. Cultures were acclimated to growth conditions for four 5-day transfers prior to measurement.

### 2.4. Carbonate chemistry manipulations

Carbon dioxide concentrations were manipulated by bubbling media with 400 µatm *p*CO_2_ or 2400 µatm *p*CO_2_ air for 48 h, and then mixing these in appropriate proportions to make 400 or 1000 µatm *p*CO_2_ growth media. The resulting CO_2_ concentrations were verified as follows: CO_2_ concentration and dissolved inorganic carbon (DIC) were calculated from TA and pH by measuring the pH of the media at 5°C (3 × 60 ml samples per flask), using CO2SYS (v. 2.1) and accounting for phosphate and silicate concentrations in the growth medium.

### 2.5. Growth rate measurements

For growth assays, three isolates were randomly chosen per mesocosm that we had uncontaminated samples from. We measured growth rates at 400 and 1000 µatm *p*CO_2_. Cultures were inoculated at 30 cells ml^−1^ in a total volume of 65 ml of f/8 medium, and placed in the incubator in a random order. Cells were counted microscopically using an Utermöhl chamber [10] at 0 and 3 days. The cell division rates were calculated as *T*_d_ = ln(*d*_2_/*d*_1_)/(Δt*ln2), where *T*_d_ is the doubling rate, *d*_1_ the initial cell density, *d*_2_ the final cell density and Δ*t* the total time for the observations. DIC-drawdown was kept below 2% during acclimation and below 1% during growth measurements. This was verified by measuring pH and as described in the Carbonate chemistry manipulations section. Cultures were growing exponentially during acclimation and growth measurements.

### 2.6. Statistical analyses

Data were analysed as a mixed model in an R environment using the nlme package [11]. The response variable is growth rate in the laboratory. The effects included are mesocosm CO_2_ level, laboratory CO_2_ level, mesocosm identity and clone. Mesocosm and laboratory CO_2_ levels were modelled as fixed effects with interaction. Mesocosm identity and clone identity were random effects, with clone nested within mesocosm identity. Note that all results (evolutionary and plastic responses) are discussed using the same statistical test in order to avoid multiple tests on the same dataset. Data are available as an online data supplement.

## 3. Results

**Figure 2.**
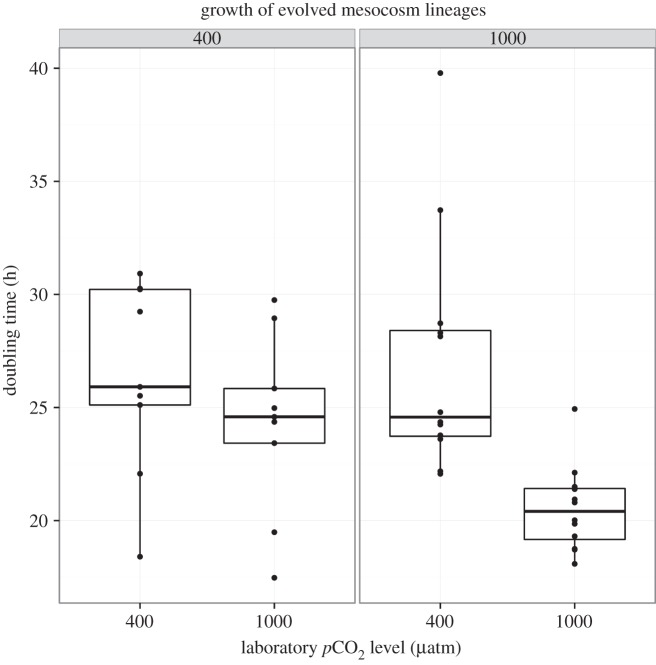
Cell division rates in hours per day for *S. marinoi* at 400 and 1000 µatm *p*CO_2_ in laboratory growth experiments. CO_2_ levels in top grey panels indicate the level of CO_2_ in the mesocosm where the lineages evolved. CO_2_ levels indicated on the bottom *x*-axis indicate CO_2_ level under which growth was measured in the laboratory. Points show cell division rates for individual lineages.

There is a direct response to selection for growth in a high CO_2_ environment in *S. marinoi* (effect of mesocosm CO_2_ × laboratory CO_2_: *t*_1,61_ =−3.45, *p*= 0.001). The direct response to selection is measured by comparing the growth of the high CO_2_-evolved lineages and ambient CO_2_-evolved lineages when grown in high CO_2_ conditions in the laboratory (figure 2), and it reflects heritable differences in growth in a stable environment that are attributable to having evolved in different environments. The shorter doubling times of the high CO_2_-evolved lineages indicate that *S. marinoi* from high CO_2_ mesocosms have evolved in response to high CO_2_, and they divide about 1.3 times faster under high CO_2_ laboratory conditions than do lineages from control mesocosms. The doubling rate for lineages from high CO_2_ mesocosms is 20.53 ± 1.87 h (mean ± s.d.) at high CO_2_, while the doubling rates for lineages from control mesocosms is 24.32 ± 3.89 h at high CO_2_.

A plastic response is a change in phenotype of a single genotype to environmental change. Here, this corresponds to the difference in growth rates of a single isolate when it is grown in ambient versus high CO_2_ conditions in the laboratory, and reflects the ability of lineages to respond to changes in CO_2_. Overall, the plastic response to short-term changes in CO_2_ levels is to increase growth rates (same analysis as above; effect of laboratory CO_2_ level *t*_1,61_ =−2.19, *p* = 0.0318). This is driven by the responses of the lineages evolved in the high CO_2_ mesocosms (interaction between laboratory and mesocosm CO_2_ levels *t*_1,61_ =−3.45, *p* = 0.001; figure 2). While isolates of *S. marinoi* evolved in control mesocosms do not show a plastic growth response to CO_2_ enrichment, lineages evolved in the high CO_2_ mesocosms do. This shows that the plastic response to rapid changes in CO_2_ has evolved in the high CO_2_ mesocosms. Full model output is in the electronic supplementary material, appendix.

## 4. Discussion

Experimental evolution allows researchers to watch evolution in real time, and connect evolutionary responses to environmental drivers. Here, we show that microbial evolution experiments can be carried out in enclosed natural plankton communities, where experimental design and measures of evolutionary responses are the same as in laboratory experiments. These *in situ* experiments can be directly compared with laboratory experiments to link general mechanisms to particular outcomes.

Our focal species, the marine diatom *S. marinoi,* evolved in response to growth under high CO_2_ conditions for over 100 days as part of an enclosed microbial community. Both the growth rate at high CO_2_ and the plastic response to changes in CO_2_ levels evolved. The direct response to selection was large, with lineages evolved at high CO_2_ having a 1.3× growth advantage over lineages from the control mesocosms when both were grown at high CO_2_ under laboratory conditions. Since the evolutionary response to selection is to increase growth, it is likely to be adaptive, or part of a more complex phenotypic change that is, on balance, adaptive [1]. While our results show unambiguously that evolution occurred in response to high CO_2_, the fitness advantage associated with it within the mesocosms cannot be reasonably extrapolated from growth rates in the laboratory. That being said, if fitness were determined entirely by growth rate, this would translate into about a 33% fitness advantage. However, since growth is not the only component of fitness, this is likely to be an overestimate, especially if faster growing lineages are more likely to be grazed. Because high- and control-CO_2_ mesocosms also differ in their communities [12] and abiotic environment [13] as a result of the CO_2_ manipulation, we cannot say how much of the evolutionary response to CO_2_ enrichment is directly driven by CO_2_ versus indirectly. A parallel laboratory experiment where *S. marinoi* evolved in environments that differ only in CO_2_ levels (e.g. [3]) would be needed to partition the evolutionary response into components attributable to direct and indirect drivers.

Our results raise the possibility that local changes in CO_2_ levels could drive adaptation in local populations [14]. Interestingly, the asymmetry in the responses of the diatoms from the control and high CO_2_ mesocosms, where the high CO_2_-evolved lineages outgrow the control lineages at high CO_2_, but the control lineages do not outgrow the high CO_2_-evolved lineages at control levels of CO_2_, has been seen in some evolution experiments using high CO_2_ as a driver for phytoplankton evolution, though in other cases, high CO_2_-evolved lineages grew poorly or died at ambient CO_2_ (reviewed in [3]). Elevated CO_2_ may be able to drive local adaptation even if increases in growth rates are transient or absent, since marine picoplankton evolved for hundreds of generations in a high CO_2_ environment maintained an increase in competitive ability even when they did not show increased growth in the absence of competitors in laboratory high CO_2_ environments [15].

Previous studies show that plastic responses to CO_2_ enrichment are idiosyncratic between, and even within, diatom species [16], reporting that *Skeletonema* spp. can respond plastically to changes in CO_2_ by increasing growth [17] or not [16]. However, the composition of synthetic [18] and natural [19] diatom assemblages changes in response to CO_2_ enrichment, indicating that shifts in relative fitness can be large enough to allow evolution in such assemblages. We find that even though the plastic response to CO_2_ enrichment in *S. marinoi* isolated from control mesocosms is absent, lineages evolved in high CO_2_ mesocosms both respond plastically to CO_2_ enrichment and grow faster at high CO_2_. This is in line with studies in green algae showing that more plastic lineages are likely to be selected in novel environments [15]. The maximum number of generations of *S. marinoi* possible in the mesocosm experiment was approximately 100, making it unlikely that novel mutations fuelled evolution here. Because dominant mutations of very large effect could have had time to fix had they arisen early in the mesocosm experiment, we cannot rule out the possibility that novel genetic variation arose during the mesocosm experiment. However, our data suggest that it is more likely that natural selection acted predominantly on pre-existing variation, favouring more plastic genotypes in the high CO_2_ environment and less plastic genotypes in the control environment. Our reasoning is that the fastest-growing high CO_2_-evolved lineages are within the range of the control-evolved lineages, even though the average growth rate is faster. In addition, based on the sampling effort required to do this study, populations of *S. marinoi* were relatively small in the mesocosms, meaning that the supply of novel mutations would have also been low. This, alongside the variation seen among lineages in terms of plastic responses, suggests that there is substantial within-population variation in plastic responses to changes in CO_2_ in this species.

Ocean change research has made the strongest progress in recent years at the level of single species or strains with respect to their plastic (short-term) responses to single environmental changes. It is, however, the evolutionary (long-term) response of natural communities to a multitude of environmental alterations that we need to understand to make reliable predictions of future changes in marine ecosystems. Providing this information by stepping up from single to multiple drivers, from single strains to communities and ecosystems, and from plastic to evolutionary responses is a major challenge. Using mesocosm studies for experimental evolution offers a way to investigate evolutionary outcomes in natural populations that is directly comparable with laboratory evolution experiments, linking evolution in single species and community experiments. This study shows that investigating evolutionary adaptation at the community level in near-natural environmental settings is feasible and that approaches such as the one taken here will help paint a more realistic picture of the future of ocean ecosystems.

## Supplementary Material

Appendix.doc

## Supplementary Material

datasupplement
