# Supplementary material for: Experimental evolution gone wild
Source: J R Soc Interface. 2015 May 6;12(106):20150056. doi: 10.1098/rsif.2015.0056 (PMC4424681; doi:10.1098/rsif.2015.0056)
Supplement: Appendix.doc [file rsif20150056supp1.doc]

Appendix.

*Statistical model and model output.*

Data were analysed using a mixed model using the lme package (CITATION). The model is as follows:

lme(doublingtime ~ mesocosm.CO2*lab.CO2, random = ~1|mesocosm.CO2/mesocosmID/clone, data=growthdata)]]

Verbally, the response variable is the doubling time. Fixed effects in the model are:

mesocosm.CO2 = mesocosm CO_2_ levels under which the isolates evolved.

lab.CO2 = laboratory CO_2_ levels under which the growth assay was carried out.

Random effects in the model are: clone nested within mesocosm ID nested within mesocosm CO_2_ level.

clone = the specific clone in question

mesocosm ID = the particular mesocosm from which a clone was isolated

mesocosm CO2 = the CO_2_ level of a particular mesocosm from which a clone was isolated.

Model output:

Fixed effects:

Value Std.Error DF t-value p-value

(Intercept) 26.406528 1.7408462 61 15.168789 0.0000

mesocosm.CO2 0.569007 2.3766682 0 0.239414 NaN

lab.CO2 -2.090461 0.9515268 61 -2.196954 0.0318

mesocosm.CO2:lab.CO2 -4.350546 1.2587517 61 -3.456238 0.0010

Random effects:

mesocosm.CO2; standard deviation = 1.174900 (intercept)

mesocosmID in mesocosm.CO2; standard deviation = 0.000377 (intercept)

clone in mesocosmID in mesocosm.CO2; standard deviation = 3.2828 (intercept), 2.8546 (residual)
